# Improving the thermal conductivity of epoxy composites using a combustion-synthesized aggregated β-Si_3_N_4_ filler with randomly oriented grains

**DOI:** 10.1038/s41598-020-71745-w

**Published:** 2020-09-10

**Authors:** Akihiro Shimamura, Yuji Hotta, Hideki Hyuga, Mikinori Hotta, Kiyoshi Hirao

**Affiliations:** National Institute of Advanced Industrial Science and Technology, 2266-98 Anagahora, Shimo-Shidami, Moriyama-ku, Nagoya, 463-8560 Japan

**Keywords:** Engineering, Materials science

## Abstract

Electrically insulating and thermally conductive polymer matrix composites are desirable for industry applications as they improve the reliability of high-performance electronic devices, particularly via heat dissipation in devices loaded with several electronic components. In this study, an aggregated β-Si_3_N_4_ filler with randomly oriented grains was produced via combustion synthesis to improve the thermal conductivity of epoxy composites. The thermal conductivities of the prepared composites were investigated as a function of the filler content, and the values were compared to those of composites loaded with commercial β-Si_3_N_4_ (non-aggregated)_._ Negligible difference was observed in the thermal conductivities of both types of composites when the Si_3_N_4_ content was below 40 vol%; however, above 40 vol%, the aggregated β-Si_3_N_4_ filler-loaded composites showed higher thermal conductivities than the commercial β-Si_3_N_4_-loaded composites. The aggregated β-Si_3_N_4_ filler-loaded composites exhibited isotropic thermal conductivities with a maximum value of 4.7 W m^−1^ K^−1^ at 53 vol% filler content, which is approximately 2.4 times higher than that of the commercial β-Si_3_N_4_-loaded composites, thereby suggesting that the morphology of the aggregated filler would be more efficient than that of the commonly used non-aggregated filler in enhancing the thermal conductivity of a polymer matrix composite.

## Introduction

Heat dissipation in electronic devices has become increasingly important because the packing densities of these devices constantly increase, resulting in high heat generation in a compactly packed space. To improve heat dissipation, electrically insulating materials with high thermal conductivities are desired. Typically, epoxy resin is used as an insulating material for general electronic devices owing to its moderate flexibility compared to other thermosetting resins, such as phenolic, bismaleimide, and cyanate ester resins. However, its thermal conductivity is very low, typically ~ 0.2 W m^−1^ K^−1^^1^. One evident approach to improve the performance of the epoxy resin would be to improve its thermal conductivity. However, the preparation of intrinsically thermally conductive epoxy resin is cumbersome, difficult, and relatively expensive; therefore, it is unsuitable for commercial purposes.

Thermally conductive ceramic/polymer composites present new possibilities for industrial applications owing to their light weight, corrosion resistance, flexibility, and superior heat dissipation capabilities. Therefore, loading epoxy resin with a ceramic powder possessing high thermal conductivity, such as silicon nitride (Si_3_N_4_)^2,3^, aluminum nitride (AlN)^4–7^, and boron nitride (BN)^8–10^, can be considered a simpler alternative for improving the thermal conductivity of epoxy composites. BN possesses a hexagonal crystal structure and exhibits a layer-like structure with a high aspect ratio. Such morphology makes it difficult to obtain a uniform dispersion of BN grains in a composite material, and the resulting composite loaded with BN tends to have an anisotropic thermal conductivity owing to its high aspect ratio^11–14^. AlN and Si_3_N_4_ are easily available commercially compared to BN. However, the drawbacks of AlN, such as high reactivity with moisture, cannot be disregarded when producing composites with high thermal conductivity. Si_3_N_4_ is chemically stable, and it has two crystal phases: α-type and β-type. β-Si_3_N_4_ has higher thermal conductivity than α-Si_3_N_4_. Theoretical thermal conductivity of a single β-Si_3_N_4_ crystal along the a and c axes are 170 W m^−1^ K^−1^ and 450 W m^−1^ K^−1^^15^, respectively, which indicates that β-Si_3_N_4_, along the c-axis, is theoretically comparable to BN and AlN in terms of theoretical thermal conductivity. However, compared to studies on AlN- and BN-loaded epoxy composites, fewer reports have been published on β-Si_3_N_4_-loaded epoxy composites. This may be attributed to the low thermal conductivity of commercially available β-Si_3_N_4_, arising from the lower degrees of crystallinity and oxygen impurities that densify at lower sintering temperatures.

Combustion synthesis of ceramics is another energy- and time-saving approach that uses an exothermic reaction to produce a variety of ceramic products relatively quickly. Additionally, the combustion synthesis process is cost-efficient and is not as susceptible to contamination compared to conventional processes^16,17^. Hirao et al. used combustion synthesis for producing β-Si_3_N_4_ bulk product, which was then crushed to obtain single grains of β-Si_3_N_4_. The ground powder possessed a rod-like shape owing to the extended unidirectional crystal growth during the combustion reaction^18^. The rod-like shape is expected to potentially enhance the thermal conductivity of the composite because of the formation of long pathways of thermal conductivity along the longitudinal direction^19^. Moreover, the rod-like grains are likely to be oriented according to the press molding direction, causing the thermal conductivity to become anisotropic, as reported in another study^19^. Currently, only a few studies in the literature have focused on the thermal conductivity of epoxy composites loaded with combustion-synthesized Si_3_N_4_.

In this study, we produced Si_3_N_4_ bulk product using combustion synthesis. The combustion product was then moderately ground to obtain an aggregate Si_3_N_4_ filler. Next, the thermal conductivity of the composites prepared by dispersing the aggregate Si_3_N_4_ filler in various proportions was evaluated. The thermal conductivity of this composite was also compared to that of the previously reported commercial β-Si_3_N_4_-loaded composite^20^. The microstructures of the composites and the dispersion state of Si_3_N_4_ in the matrix as a function of Si_3_N_4_ filler content were also studied.

## Experimental section

### Combustion synthesis of aggregated β-Si_3_N_4_ filler

**Figure 1:**
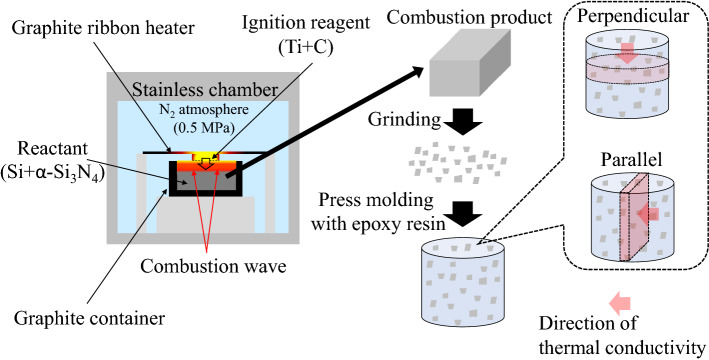
Combustion synthesis process and composite formation.

Si powders (#600, Yamaishi Metal Co., Ltd., Japan) and α-Si_3_N_4_ (SN-E10, Ube Industries, Ltd., Japan; as diluent powder) were ball-milled together for 12 h in an ethanol-filled polyethylene container using sintered Si_3_N_4_ balls. The mixture was filtered and then dried in vacuo at 75 °C. The obtained powder was subsequently sifted through a 250-μm sieve and was filled in a thin and highly porous graphite container to prevent the flow of nitrogen from entering the reaction chamber. Figure 1 illustrates the combustion synthesis of Si_3_N_4_ and preparation of the composite. The combustion reaction was conducted in a high-pressure stainless-steel chamber vessel with a graphite ribbon heater attached to the inside of the chamber. The combustion reaction was initiated by igniting a titanium/carbon powder compact placed on top of the mixture kept in a graphite container with a carbon ribbon heater. The combustion experiment was then conducted at 0.5-MPa nitrogen pressure. Following the combustion reaction, the bulk product was ground to powder using a mortar and then sieved through a 250-μm sieve. Hereinafter, the sieved powder product is referred to as combustion-synthesized aggregated β-Si_3_N_4_ (CA-SN).

### Preparation of the composite material

CA-SN was mixed with the epoxy resin comprising bisphenol A (EPICRON855, DIC Co. Ltd., Japan) and a curing agent (JER CURE ST12, Mitsubishi Chemical Co. Ltd., Japan). The resultant mixture was cured at 120 °C for 2 h in a metal mold under 70 MPa to fabricate the composites. The CA-SN filler content was varied from 30 to 70 vol% in the composites. The composites were cut in two different directions “perpendicular” and “parallel” as shown in Fig. 1, to measure the direction-dependent anisotropy of the thermal conductivity.

### Characterization

The crystalline structure of the combustion product was characterized via X-ray diffraction (XRD: RINT-2500, Rigaku Co. Ltd., Japan). The specific surface area of the obtained powder was measured using the Brunauer, Emmett, and Teller method with nitrogen as the adsorbing gas (QDS-30, Quantachrom, USA). The amount of oxygen impurities dissolved in the CA-SN filler was determined using a hot-gas extraction analyzer (EMGA-820, Horiba Co. Ltd., Japan). The particle size distribution of the CA-SN filler was measured using a laser-diffraction particle size analyzer (Horiba LA-920, Horiba Ltd., Japan). The powder product was dispersed in water by applying ultrasonic treatment before the measurement was taken. The morphologies of the CA-SN filler and the composites were evaluated using a scanning electron microscope (SEM; JSM-5600, JEOL, Japan). The relative densities of the composites were measured using the Archimedes’ method. The thermogravimetric (TG8120, Rigaku Co., Japan) analyses of the composites were carried out under air flow at a heating rate of 10 °C min^−1^ from room temperature to 600 °C to determine the precise amount of Si_3_N_4_ in the composites. We selected this specific temperature because we had previously confirmed that although the epoxy resin completely burned out at 600 °C, the Si_3_N_4_ remained stable at this temperature^20^. The weight loss in the composite was observed until 600 °C owing to this burn-out process of the epoxy resin. The CA-SN filler content was calculated from the residual weight at 600 °C. We adapted the laser flash method to measure the thermal diffusivity (Model TC-7000, ULVAC, USA) along the perpendicular and parallel directions, as shown in Fig. 1 because this method can directly measure thermal diffusivity of the sample depending on the direction^19,21^. The thermal conductivity of the composites was calculated using thermal diffusivity, specific heat, and bulk density. The specific heat of the composites was calculated from the theoretical values corresponding to Si_3_N_4_ (0.68 J g^−1^ K^−1^) and the epoxy resin (1.1 J g^−1^ K^−1^) using the rule of mixtures.

## Result and discussion

### Characterization of the combustion-synthesized aggregated β-Si_3_N_4_ filler

**Figure 2:**
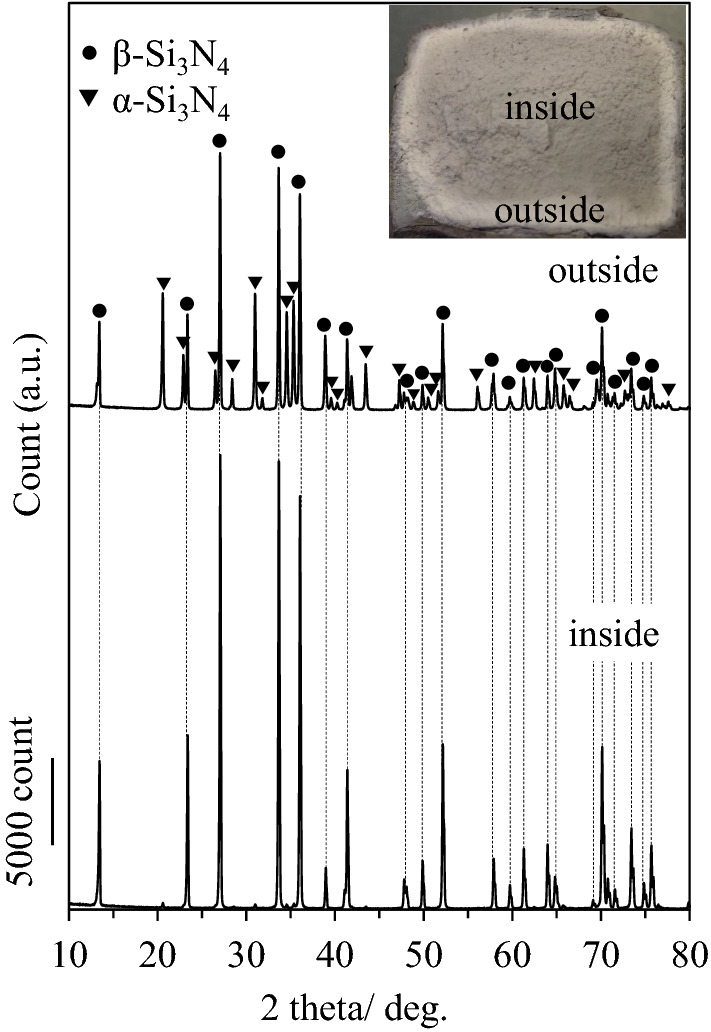
XRD patterns and cross-sectional images of the inner and outer portions of the combustion-synthesized Si_3_N_4_ product.

The XRD patterns of the CA-SN fillers are shown in Fig. 2, the inset of which shows the cross-sectional image of the CA-SN bulk product. The outer layer of this bulk product is white, whereas the inner portion appears to be gray. The XRD patterns of the inner and outer layers of the bulk product are displayed at the bottom and top, respectively. The diffraction peaks of the inner portion of the product were mainly attributed to β-Si_3_N_4_, whereas the diffraction peaks of the outer portion of the product were attributed to both α-Si_3_N_4_ and β-Si_3_N_4_. No silicon and silica peaks were observed in the inner or outer layers of the product, indicating that the silicon powder successfully reacted with nitrogen to form Si_3_N_4_ during the combustion process. The α-Si_3_N_4_ and β-Si_3_N_4_ phases are the low- and high-temperature phases, respectively. The transformation of α phase to its β counterpart occurs at temperatures above 1,300 °C^15^. Hirao et al. reported that α-Si_3_N_4_ is present in the surface layer of the combustion products because the combustion temperature of the surface layer was lower than that of the interior owing to the thermal loss from the side surface^18^. Because α-Si_3_N_4_ has lower thermal conductivity than β-Si_3_N_4_^15^, only the inner portion of the bulk product was used in the following discussion.

**Figure 3:**
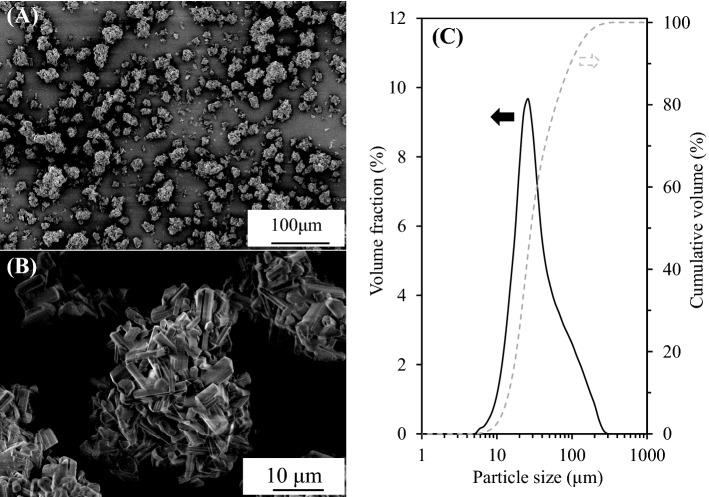
SEM images with particle size distribution of combustion-synthesized aggregated β-Si_3_N_4_ filler: (**A**) lower magnification SEM images, (**B**) higher magnification SEM images, and (**C**) particle size distribution.

The SEM images of the CA-SN filler are shown in Fig. 3A,B, respectively. The CA-SN filler shows aggregated particle of several tens of micrometers in size (Fig. 3A). The highly magnified image of the CA-SN filler (Fig. 3B) shows that the aggregate was composed of rod-like individual grains that were identified as typical, randomly oriented β-Si_3_N_4_ grains. β-Si_3_N_4_ grains grow preferentially into a typically hexagonal rod-like shape, attributed to the much faster growth rate along the [001] (or c-axis) direction than along the [210] direction, namely anisotropic or elongated grain growth. Therefore, the Lotgering orientation factor (f_00l_) was used to evaluate the degree of c-axis orientation in β-Si_3_N_4_ bulk, according to Eq. (1)^22^:

1 $$f=\frac{P-{P}_{0}}{1-{P}_{0}}$$

where P and P_0_ = ΣI(001)/Σ(*hkl*) denote the intensities of the (hkl) reflections in the range of 2θ from 10° to 70°. The value of P was calculated from the ratio of the sum of the (00 l) intensity to that of all (hkl) intensities, and the value of P_0_ was calculated from the standard data of the JCPDS card (No. 3-1160). The Lotgering orientation factor was calculated to be 0.005 from the XRD pattern of CA-SN (Fig. 2, inset). This value was nearly zero, indicating that the grains are not oriented in one direction. It was also confirmed that the elongated β-Si_3_N_4_ grains were randomly oriented in the CA-SN filler.

Figure 3C shows the particle size distribution of the CA-SN filler obtained after ultrasonic dispersion (as mentioned in “Experimental section”). The CA-SN filler has an average particle size of 26 μm, which is significantly larger than the size of an individual β-Si_3_N_4_ grain. Furthermore, the aggregated structure is retained even after sonication, indicating that the individual β-Si_3_N_4_ grains were connected. Table 1 shows the characterization results of the CA-SN and commercial β-Si_3_N_4_ powders (SN-F1, Denka Co. Ltd, Japan) used as filler in a previous study^20^ (SEM image of the commercial β-Si_3_N_4_ powder can be found as Supplementary Fig. S1A). The mean diameters of the CA-SN and commercial β-Si_3_N_4_ powders were 26 μm and 4.5 μm, respectively. The specific surface areas of the CA-SN and commercial β-Si_3_N_4_ powders were 3.0 m^2^ g^−1^ and 4.1 m^2^ g^−1^, respectively. In general, the specific surface area increases with decreasing particle size. However, the specific surface areas of both powders were observed to be similar although the particle sizes differed by a factor of 5. This indicates that the individual β-Si_3_N_4_ grains are partially sintered with each other to form an aggregate structure, suggesting that it is this aggregate structure that allows the heat to pass more efficiently through the CA-SN filler compared to the commercially available β-Si_3_N_4_ powder. Furthermore, the total oxygen contents of the CA-SN and commercial β-Si_3_N_4_ powders were 0.29% and 1.83%, respectively, indicating that the oxygen content in β-Si_3_N_4_ was significantly reduced by combustion synthesis. It has been reported that the thermal conductivity of Si_3_N_4_ increases with a decrease in oxygen content^21^. Evidently, the individual β-Si_3_N_4_ grains in the CA-SN filler showed a higher thermal conductivity compared to the commercially available β-Si_3_N_4_. Therefore, we can conclude that the CA-SN filler formed large aggregates (few tens of microns in size) with random orientations of β-Si_3_N_4_ grains and a low oxygen content.

**Table 1:** Characterization of the combustion-synthesized aggregated β-Si_3_N_4_ and commercial β-Si_3_N_4_.

	Mean diameter (μm)	Specific surface area (m^2^ g^−1^)	Total oxygen content (%)
Combustion-synthesized aggregated β-Si_3_N_4_	26	3.0	0.29
Commercial β-Si_3_N_4_	4.5	4.0	1.81

### Characterization of epoxy composite loaded with combustion-synthesized aggregated β-Si_3_N_4_ filler

**Figure 4:**
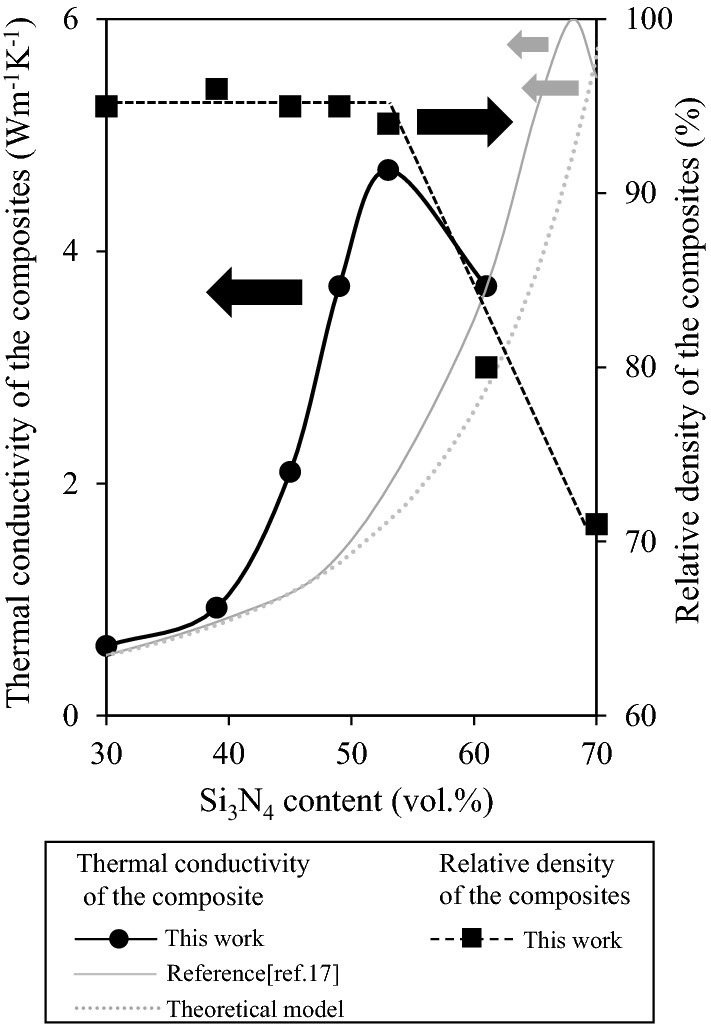
Thermal conductivity and relative density of the combustion-synthesized aggregated β-Si_3_N_4_ filler-loaded epoxy composites as a function of Si_3_N_4_ powder content.

The thermal conductivity in the perpendicular direction and the relative density of the CA-SN-loaded composites as a function of CA-SN filler content are plotted as black solid circles and black solid squares, respectively, in Fig. 4. The filler content in Fig. 4 was calculated from the weight–loss curve for up to 600 °C because the β-Si_3_N_4_ was stable until 600 °C and only the epoxy resin burned out near 600 °C (typical weight–loss curve at 53 vol% can be found as Supplementary Fig. S2). The gray solid curve shows the thermal conductivity of the commercial β-Si_3_N_4_/epoxy composites obtained using our previous work as a Ref.^20^, wherein the thermal conductivity was also measured in the perpendicular direction. The gray dotted curve represents the theoretical thermal conductivity of β-Si_3_N_4_/epoxy composite using the Bruggeman model. The experimental value of the thermal conductivity of CA-SN composites showed a gradual increase with increase in powder content of up to 40 vol%, and it increased rapidly up to 53 vol%. The maximum thermal conductivity of 4.7 W m^−1^ K^−1^ was observed at 53 vol%. Additionally, we measured the thermal conductivity in the parallel direction at 53 vol%, yielding a value of 4.8 W m^−1^ K^−1^. The similarity in the measured values of the thermal conductivity along the parallel and perpendicular directions indicates the isotropic nature of the CA-SN composite’s thermal conductivity. After reaching 53 vol%, thermal conductivity decreased with further increase in the powder content. The composites show a higher relative density (~ 95%) until the volume fraction of the CA-SN filler reaches 53 vol%. A further increase in the powder volume fraction to 61 vol% and 70 vol% resulted in a decrease in the relative density of the composites to 80% and 71%, respectively. Such a decrease in the relative density was attributed to the incomplete molding of the composite. Compared to the commercial β-Si_3_N_4_-loaded composites from our previous study^20^, the thermal conductivity values of the CA-SN composites were similar to that of commercial β-Si_3_N_4_ composites until 40 vol%; however, above 40 vol%, the thermal conductivity of the CA-SN composites was higher than that of commercial β-Si_3_N_4_ composites. The thermal conductivities of the commercial β-Si_3_N_4_ composite at 53 vol% (Si_3_N_4_) along the perpendicular and parallel directions were 1.9 W m^−1^ K^−1^ and 2.1 W m^−1^ K^−1^, respectively. As the commercial β-Si_3_N_4_ was in the form of crushed powder (see Supplementary Fig. S1A), the thermal conductivity difference between the parallel and perpendicular directions was moderate. At 53 vol%, the thermal conductivity of the CA-SN composite was approximately 2.4 times higher than that of the commercial β-Si_3_N_4_ composite. These experimental results were compared to the theoretical thermal conductivity obtained using the Bruggeman model, shown as the gray dotted curve in Fig. 4. The Bruggeman model is based on the hypothesis that the spherical particles are distributed homogeneously in a matrix and that they are separated from each other (i.e., there is no contact between the particles)^23^. Using this model, the thermal conductivity of a composite is described in Eq. (2):

2 $$1-\frac{\varphi }{100}=\frac{\left({\lambda }_{c}-{\lambda }_{f}\right)}{\left({\lambda }_{r}-{\lambda }_{f}\right)}\times {\left(\frac{{\lambda }_{r}}{{\lambda }_{c}}\right)}^{1/3}$$

where *λ*_*f*_ is the thermal conductivity of β-Si_3_N_4_ (W m^−1^ K^−1^), *λ*_*r*_ is the thermal conductivity of the epoxy resin (W m^−1^ K^−1^), *λ*_*c*_ is the thermal conductivity of the composite (W m^−1^ K^−1^), and *φ* is the content of β-Si_3_N_4_ (vol%). The thermal conductivity of the epoxy resin (*λ*_*r*_) is 0.2 W m^−1^ K^−1^ based on our experimental results, and the thermal conductivity of *λ*_*f*_ for β-Si_3_N_4_ is 106 W m^−1^ K^−1^, which was estimated by calculating the simple arithmetic mean of thermal conductivity values measured along three coordinate axes based on Li et al.’s work^15^. The experimental thermal conductivity of the CA-SN composites showed a good fit with the theoretical values calculated using the Bruggeman model below 40 vol%, which suggests that the CA-SN fillers are homogeneously distributed in the epoxy resin matrix (Fig. 4). Furthermore, the CA-SN filler has a minimal effect on the thermal conductivity of the composite for the lower filler content volume percentages because the heat flow through the Si_3_N_4_ filler is hindered by the epoxy resin owing to the low thermal conductivity (~ 0.2 W m^−1^ K^−1^) of the resin matrix. In the CA-SN filler range of 40–53 vol%, the theoretical model deviates significantly from the experimental values of thermal conductivity; the higher experimental values may be attributed to the formation of a percolation network among the CA-SN fillers. The percolation network is a system where the powder particles are in physical contact, forming a network in the matrix^24,25^. The thermal conductivity of the composites is dominated by the conduction path formed in the matrix. As is evident from Fig. 4, thermal conductivity increases rapidly with an increase in the powder content up to 53 vol% due to the formation of the thermal conduction path in the matrix. This behavior of the CA-SN-loaded composites is clearly distinct from that of commercial β-Si_3_N_4_-loaded composites.

**Figure 5:**
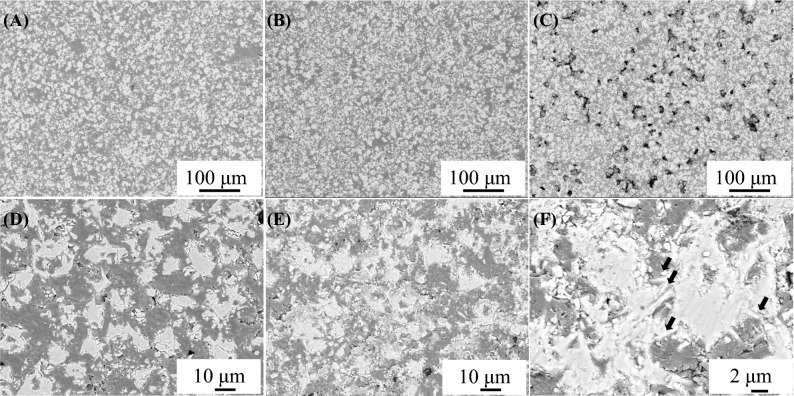
SEM images of the combustion-synthesized β-Si3N4/epoxy composites with BSEs at 40 vol% (**A**, **D**), 53 vol% (**B**, **E**, **F**), and 62 vol% (**C**). The lighter regions correspond to the combustion-synthesized β-Si_3_N_4_, and the darker regions correspond to the epoxy resin matrix.

**Figure 6:**
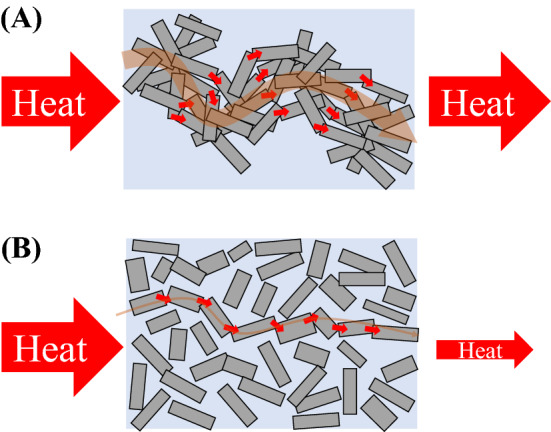
Schematic demonstration of the effect of morphology of (**A**) combustion-synthesized β-Si_3_N_4_ filler and (**B**) commercial β-Si_3_N_4_ filler on the thermal conductivity of composites.

Figure 5 shows the SEM images with backscattered electrons (BSEs) of the CA-SN composites at 40 vol% (Fig. 5A,D), 53 vol% (Fig. 5B,E,F), and 62 vol% (Fig. 5C) CA-SN filler content.(For comparison, SEM image with BSE of commercial β-Si_3_N_4_ loaded composite at 53 vol.% filler content can be found in Supplementary Fig. S1B). The small voids observed in Fig. 5A,B,D–F, were due to the shedding of Si_3_N_4_ grains when polishing the surface of the composite for SEM observation. As shown in Fig. 4, the relative density of the composites was high, namely ~ 96% until 53 vol% of the CA-SN filler content; hence, the composites were highly dense. The SEM images shown in Fig. 5A–C were captured at lower magnification, whereas the images in Fig. 5D–F were captured at higher magnification. The BSE images provide information regarding the material composition because heavier atoms (with high atomic numbers) scatter electrons more effectively than the lighter atoms (with low atomic numbers). In the BSE images of the CA-SN composites shown in Fig. 5, the CA-SN fillers and epoxy resin appear as lighter and darker regions, respectively. The low-magnification SEM images show that the CA-SN filler was uniformly distributed in the epoxy resin matrix of the composite at 40 vol% and 53 vol% CA-SN filler content; however, larger voids were observed in the composite at 62 vol% powder content owing to the incomplete molding of the composite. The highly magnified SEM image (Fig. 5D) shows that the CA-SN fillers were relatively mono-dispersed in the matrix at 40 vol%, whereas the CA-SN fillers appear to be in contact with each other at 53 vol% (Fig. 5E), thereby confirming the formation of a percolation network among the CA-SN filler particles. Figure 5F shows the detailed morphology of the percolation network formed among the CA-SN filler particles. The CA-SN filler particles were in contact with each other in the composite. In some parts of the CA-SN filler, the rod-like β-Si_3_N_4_ grains (indicated using black arrows in Fig. 5F) between the CA-SN fillers appeared slightly to enter the fillers instead of simply contacting at the points, thereby making the structure intricate. Table 2 shows the summary of the reported thermal conductivities of epoxy composite loaded with Si_3_N_4_ and AlN. While Si_3_N_4_ and AlN have similar particle sizes as CA-SN, AlN is known to have a higher thermal conductivity compared to Si_3_N_4_; for example, the thermal conductivity of commercial AlN substrate was measured to be approximately twice as high as that of commercial Si_3_N_4_ substrate^26^. The loaded content of AlN was ~ 55–60 vol%, which was slightly higher than that of the CA-SN composites (53 vol%) at the maximum value of thermal conductivity. The reported thermal conductivity values of Si_3_N_4_ and AlN composites were ~ 3.0 W m^−1^ K^−1^ and ~ 3.4–4.0 W m^−1^ K^−1^, respectively. Compared to Si_3_N_4_ and AlN, as per the data listed in Table 2, CA-SN provides the best results with regard to the enhancement of thermal conductivity of the epoxy composite. This improvement in the thermal conductivity of our samples may be attributed to two major reasons: the oxygen content and morphology of the powdered particles. As shown in Table 1, CA-SN has a lower oxygen content compared to commercial Si_3_N_4_. The thermal conductivity of Si_3_N_4_ increases with a decrease in oxygen content^21^. However, the thermal conductivity of the CA-SN-loaded composite was higher than the reported values for the AlN-loaded composites. Therefore, this enhancement cannot be only explained in the context of lower oxygen content. Hence, the morphology of the powdered (filler) particles would be a more significant factor in explaining this behavior. Figure 6 presents the schematic view of the composite loaded with CA-SN fillers (Fig. 6A) and commercial Si_3_N_4_ fillers (Fig. 6B). In the commercial Si_3_N_4_-loaded composites (Fig. 6B), the β-Si_3_N_4_ fillers came in contact with each other when the percolation network among them was formed, leading to an increase in the thermal conductivity of the composite. However, the CA-SN filler is an aggregate filler wherein the rod-like β-Si_3_N_4_ grains were partially sintered with each other (Fig. 6A). This morphology allows the heat to be conducted even more efficiently between the β-Si_3_N_4_ grains in the CA-SN filler compared to commercial β-Si_3_N_4_ filler, where they are only in contact. Furthermore, the individual β-Si_3_N_4_ grains in the CA-SN filler have a lower impurity oxygen content, suggesting that CA-SN has a higher thermal conductivity than commercial β-Si_3_N_4_.

**Table 2:** Summary of the previously reported thermal conductivities of epoxy composites loaded with Si_3_N_4_ and AlN.

Thermal conductivity (W m^−1^ K^−1^)	Ceramic type [average particle size (μm)]	Content of ceramic (vol%)	References
~ 3.0	Si_3_N_4_ (D_50_: 4 μm)	60	^2^
~ 3.4	AlN (D_50_: 6 μm)	57	^4^
~ 3.8	AlN (D_50_: 7 μm)	55	^5^
~ 4.0	AlN (D_50_: 10 μm)	60	^6^

## Conclusion

In this study, we synthesized an aggregated β-Si_3_N_4_ filler with randomly oriented grains via combustion synthesis and evaluated the enhancement in thermal conductivity of the epoxy composite loaded with it. An aggregated filler composed of randomly oriented rod-like β-Si_3_N_4_ structures with a low oxygen content was obtained using the combustion reaction. The combustion-synthesized β-Si_3_N_4_-loaded epoxy composites showed an isotropic, high thermal conductivity of 4.7 W m^−1^ K^−1^ at 53 vol% filler content, which is approximately 2.4 times higher than that of commercial β-Si_3_N_4_-loaded composites. This behavior is attributed to the formation of a percolation network among the β-Si_3_N_4_ aggregate filler particles in the epoxy resin matrix. Our findings suggest that the combustion-synthesized β-Si_3_N_4_ filler is more efficient in enhancing the thermal conductivity of a polymer matrix composite than the commonly used commercial β-Si_3_N_4_ filler.

## Supplementary information

Supplementary Figures.

## References

[1] Guo Y, Ruan K, Shi X, Yang X, Gu J (2020). Factors affecting thermal conductivities of the polymers and polymer composites: A review. Compos. Sci. Technol..

[2] Shi Z, Fu R, Agathopoulos S, Gu X, Zhao W (2012). Thermal conductivity and fire resistance of epoxy molding compounds filled with Si_3_N_4_ and Al(OH)_3_. Mater. Des..

[3] He H, Fu R, Han Y, Shen Y, Song X (2007). Thermal conductivity of ceramic particle filled polymer composites and theoretical predictions. J. Mater. Sci..

[4] Lee E-S, Lee S-M, Shanefield DJ, Cannon WR (2008). Enhanced thermal conductivity of polymer matrix composite via high solids loading of aluminum nitride in epoxy resin. J. Am. Ceram. Soc..

[5] Xu Y, Chung DDL, Mroz C (2001). Thermally conducting aluminum nitride polymer-matrix composites. Compos. A Appl. Sci. Manuf..

[6] Nagai Y, Lai G-C (1997). Thermal conductivity of epoxy resin filled with particulate aluminum nitride powder. J. Ceram. Soc. Jpn..

[7] Bae JW, Kim W, Cho SH, Lee SH (2000). The properties of AlN-filled epoxy molding compounds by the effects of filler size distribution. J. Mater. Sci..

[8] Yung KC, Liem H (2007). Enhanced thermal conductivity of boron nitride epoxy-matrix composite through multi-modal particle size mixing. J. Appl. Polym. Sci.

[9] Zheng Z, Cox M, Li B (2018). Surface modification of hexagonal boron nitride nanomaterials: A review. J. Mater. Sci..

[10] Permal A (2016). Thermal and mechanical properties of epoxy composite filled with binary particle system of polygonal aluminum oxide and boron nitride platelets. J. Mater. Sci..

[11] Tanimoto M, Yamagata T, Miyata K, Ando S (2013). Anisotropic thermal diffusivity of hexagonal boron nitride-filled polyimide films: Effects of filler particle size, aggregation, orientation, and polymer chain rigidity. ACS Appl. Mater. Interfaces..

[12] Ma T (2020). Highly thermal conductivities, excellent mechanical robustness and flexibility, and outstanding thermal stabilities of aramid nanofiber composite papers with nacre-mimetic layered structures. ACS Appl. Mater. Interfaces..

[13] Zhang R-H (2020). Thermally conductive and insulating epoxy composites by synchronously incorporating Si-sol functionalized glass fibers and boron nitride fillers. Chin. J. Polym. Sci..

[14] Tang L (2019). Functionalized glass fibers cloth/spherical BN fillers/epoxy laminated composites with excellent thermal conductivities and electrical insulation properties. Compos. Commun..

[15] Li B (1999). Measuring the anisotropic thermal diffusivity of silicon nitride grains by thermoreflectance microscopy. J. Eur. Ceram. Soc.

[16] Hirao K, Miyamoto Y, Koizumi M (1986). Synthesis of silicon nitride by a combustion reaction under high nitrogen pressure. J. Am. Ceram. Soc.

[17] Cano IG, Borovinskaya IP, Rodriguez MA, Grachev VV (2002). Effect of dilution and porosity on self-propagating high-temperature synthesis of silicon nitride. J. Am. Ceram. Soc..

[18] Hirao, K., Miyamoto, Y. & Koizumi, M. Combustion reaction characteristics in the nitridation of silicon. *Adv. Ceramic Mater.* **2**, 780–783 (1987).

[19] Kusunose T, Yagi T, Firoz SH, Sekino T (2013). Fabrication of epoxy/silicon nitride nanowire composites and evaluation of their thermal conductivity. J. Mater. Chem. A.

[20] Shimamura A, Hotta Y, Hyuga H, Kondo N, Hirao K (2015). Effect of amounts and types of silicon nitride on thermal conductivity of Si_3_N_4_/epoxy resin composite. J. Ceram. Soc. Jpn..

[21] Kitayama M (2000). Thermal conductivity of β-Si_3_N_4_: II, effect of lattice oxygen. J. Am. Ceram. Soc..

[22] Zhu X, Sakka Y (2008). Textured silicon nitride: Processing and anisotropic properties. Sci. Technol. Adv. Mater..

[23] Bruggeman DAG (1935). Berechnung verschiedener physikalischer Konstanten von heterogenen Substanzen. I. Dielektrizitätskonstanten und Leitfähigkeiten der Mischkörper aus isotropen Substanzen. Ann. Phys..

[24] Bunde A, Dieterich W (2000). Percolation in composites. J. Electroceram..

[25] Pietrak K, Wiśniewski TS (2015). A review of models for effective thermal conductivity of composite materials. J. Power Technol..

[26] Hirao K, Zhou Y, Hyuga H, Ohji T, Kusano D (2012). High thermal conductivity silicon nitride ceramics. J. Korean Ceram. Soc..

