# Author Correction: Improving the thermal conductivity of epoxy composites using a combustion-synthesized aggregated β-Si_3_N_4_ filler with randomly oriented grains

**DOI:** 10.1038/s41598-020-80009-6

**Published:** 2020-12-22

**Authors:** Akihiro Shimamura, Yuji Hotta, Hideki Hyuga, Mikinori Hotta, Kiyoshi Hirao

**Affiliations:** National Institute of Advanced Industrial Science and Technology, 2266-98 Anagahora, Shimo-Shidami, Moriyama-ku, Nagoya, 463-8560 Japan

Correction to: *Scientific Reports* 10.1038/s41598-020-71745-w, published online 10 September 2020

This Article contains an error, where Figure 6 is incorrect. The correct version of Figure 6 appears below as Figure 1.

**Figure 1:**
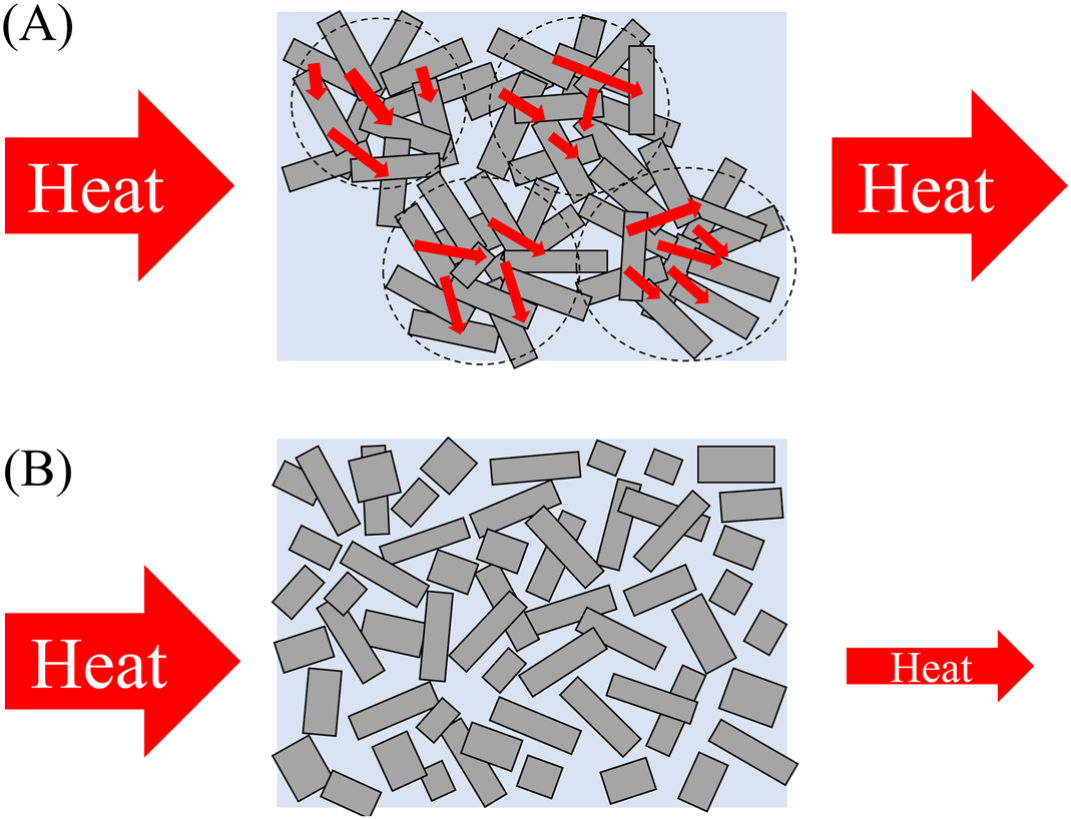
A correct version of the original Figure 6.

