# Supplementary material for: Improving the thermal conductivity of epoxy composites using a combustion-synthesized aggregated β-Si3N4 filler with randomly oriented grains
Source: Sci Rep. 2020 Sep 10;10:14926. doi: 10.1038/s41598-020-71745-w (PMC7483705; doi:10.1038/s41598-020-71745-w)
Supplement: Supplementary file 1 — Supplementary Figures. [file 41598_2020_71745_MOESM1_ESM.docx]

**Improving the thermal conductivity of epoxy composites using** **a combustion-synthesized aggregated β-Si3N4 filler with randomly oriented grains**

**Authors:**

**Akihiro Shimamur**a^1*^, ORCID code: https://orcid.org/0000-0001-7254-4355

Yuji Hotta^1^, ORCID code: https://orcid.org/0000-0003-2549-4484

Hideki Hyuga^1^, ORCID code: https://orcid.org/0000-0002-6522-0421

Mikinori Hotta^1^, ORCID code: https://orcid.org/0000-0002-9250-9415

Kiyoshi Hirao^1^, ORCID code: https://orcid.org/0000-0002-5285-4230

^1^ National Institute of Advanced Industrial Science and Technology, 2266-98 Anagahora, Shimo-Shidami, Moriyama-ku, Nagoya 463-8560, Japan

*Corresponding author: [a-shimamura@aist.go.jp](mailto:a-shimamura@aist.go.jp)

Supplementary Figure S1

Figure S1 SEM image of (A) commercial β-Si_3_N_4_ powder and (B) the commercial β-Si_3_N_4_ loaded epoxy composites with 53 vol% filler content.

Supplementary Figure S2

Figure S2 Weight loss curve of the combustion-synthesized β-Si3N4 loaded epoxy composites with 53 vol% filler content.

Figure S2 Weight loss curve of the combustion synthesized aggregated β-Si_3_N_4_ loaded epoxy composite with at 53 vol% filler content.
